# Morphological variations of the brachial artery and their clinical significance: a systematic review

**DOI:** 10.1007/s00276-023-03198-5

**Published:** 2023-08-02

**Authors:** Marcin Glin, Nicol Zielinska, Kacper Ruzik, Piotr Karauda, Marko Konschake, Łukasz Olewnik

**Affiliations:** 1 Department of Anatomical Dissection and Donation, Medical University of Lodz, Lodz, Poland; 2 Department of Anatomy, Histology and Embryology, Institute of Clinical and Functional Anatomy, Medical University Innsbruck (MUI), Innsbruck, Austria

**Keywords:** Brachial artery, Superficial brachial artery, Brachioradial artery, Accessory brachial artery, Absence of brachial artery, Diameter of brachial artery

## Abstract

**Purpose:**

Morphological variations of the brachial artery are quite commonly discovered in routine dissection and have been the subject of many studies. However, there is a need for a clear classification. This work presents morphological variations of the brachial artery, based on numerous case reports and studies created for the appropriate classification and interpretation among surgeons and radiologists. It also discusses the most important clinical aspects of the given varieties.

**Methods:**

The research method is based on the combined interpretation of the researches based on numerous publications concerning both the principles of correctly classifying the described morphological variations of the brachial artery and the resulting clinical implications. This work considers atypical variations such as the presence of the superficial brachial artery, brachoradial artery, accessory brachial artery and absence of the brachial artery. Variations of the brachial artery in relation to the external and internal diameter of the vessel have also been discussed.

**Results:**

After conducting a complex analysis of the collected data, the fundamental principles for classifying such variability as superficial brachial artery, brachioradial artery and accessory brachial artery were defined. Additionally, clinical implications resulting from the above like the impact of the superficial brachial artery on the median nerve neuropathy and the positive correlation between the brachioradial artery and increased danger of incorrect transradial catheterization were demonstrated.

**Conclusions:**

The clinical implications of the atypical arterial pattern within the upper limb are crucial during the angiography and surgical procedures so the variations affect the appropriate diagnosis and surgical intervention. Hence, the knowledge about the morphological variations of the brachial artery should be constantly broadened by radiologists and surgeons to improve the accuracy and effectiveness of the treatment process.

## Introduction

The brachial artery (BA) is the main arterial trunk of the arm acting as the direct extension of the axillary artery (AA). The BA begins at the lower border of the teres major muscle and ends in the cubital fossa under cover of the bicipital aponeurosis [5]. When the BA reaches the cubital fossa, it bifurcates into two terminal branches, the radial artery (RA) and the ulnar artery (UA). The BA is located in the anterior compartment of the arm. In the proximal part, the BA is located medially to the humerus. In the distal arm, the BA courses laterally to assume a position midway between the medial epicondyle and the lateral epicondyle of the humerus [9, 23]. Before the bifurcation into RA and UA the BA crosses anteriorly to the elbow joint, where it is in close proximity to the tendon of the biceps brachii muscle.

## Branches of the BA

In addition to the above-mentioned RA and UA along its length, BA gives off branches. The deep brachial artery (DBA) or the profunda brachii artery (PBA) departs from the upper part of the BA below the inferior border of the teres major muscle, and it is the largest and highest outgoing branch [3]. It enters the posterior compartment of the arm, falls along with the radial nerve towards the radial groove and in the end the DBA bifurcates into middle collateral and radial collateral arteries [23]. Another important branch is the humeral nutrient artery. This vessel arises from the BA in the middle of the arm to penetrate the nutrient canal which is located on the surface of the humerus [14]. According to the literature, additional humeral nutrient arteries may occur [23]. The next artery that departs directly from the BA is the superior ulnar collateral artery, which is accompanied by the ulnar nerve. The superior ulnar collateral artery descends posterior to the medial epicondyle of the humerus and ends in anastomosis with the posterior ulnar recurrent artery and inferior ulnar collateral artery [23, 31]. The lowest-lying branch deviating from the BA is the inferior ulnar collateral artery. This vessel starts its course approximately 5 cm proximal to the elbow crease. Inferior ulnar collateral artery is situated anterior to the medial epicondyle of the humerus and merges with the anterior ulnar recurrent artery [23].

## Embryonic morphogenesis of the upper limb vessels

To help the clinicians comprehend the variations of the BA, it is important to refer to the upper limb vessels development during embryogenesis. The seventh intersegmental artery gives rise to an axis artery that supplies each upper limb of the embryo. The axis artery grows distally along the ventral axial line and ends in a palmar capillary plexus. The axillary artery, brachial artery, anterior interosseous artery, and deep palmar arch are formed from the main trunk of the axis artery [5, 30, 33].

## Clinical significance of the BA

From a clinical point of view, the BA plays an important role in the measurement of blood pressure: the stethoscope is placed in the region of the cubital fossa during auscultatory blood pressure measurement. This is directly due to the superficial location of the artery in relation to the skin. The BA also plays a role in oscillometric pressure measurement: the blood pressure cuff is placed at the distal and medial part of the arm around the space between the biceps brachii muscle and triceps brachii muscle.

The hand has a complex and rich vascular network, which is mostly supplied by the two terminal branches of the BA. Since the RA and UA both form the superficial and deep palmar arch, the blood supply of the hand is ensured even if one of the arteries is occluded. The Allen test is used to assess collateral blood flow to the hands and is valuable when preparing for a procedure that can disrupt blood flow in either the radial or the ulnar artery [36]. The Allen test is uncomplicated and may be performed with only a clinician’s hands. During the test, the hand is exsanguinated by telling the patient to squeeze their fist strongly. The UA and RA are occluded by manual compression, the patient relaxes the hand, and the pressure over the UA is released. Collateral flow is assessed by measuring the time required for the return of normal coloration of the skin. Return of color in less than 5 s indicates adequate collateral flow, return in 5–10 s suggests an equivocal test, and return in more than 10 s indicates inadequate collateral circulation of the blood [8].

The cubital anastomosis or the peri-articular arterial anastomoses of the elbow is an anatomical structure in the nature of vessels. This network is supplied with arterial blood from the superior and inferior ulnar collateral arteries, branches from the DBA, branches from the RA (radial recurrent artery) and branches from the UA (ulnar recurrent artery) [23]. The cubital anastomosis of the elbow is responsible for supplying the elbow joint and its supporting structures. A network of arterial vessels allows oxygenated blood to circulate around the elbow joint no matter which position the joint is in. It provides proper nutrition to the structures of the ligaments and joint capsule minimalizing the risk of insufficient vascularization.

Supracondylar fractures of the humerus are commonly observed in children. It has been documented that approximately 12% of completely displaced supracondylar fractures in the pediatric population result in vascular injuries, with the brachial artery being predominantly affected. Notable vascular complications associated with this injury include brachial artery occlusion and the potential risk of limb loss. Additionally, limb claudication may occur as another vascular complication, which can result from either the migration of a thrombus leading to restricted blood flow or inadequate repair of the brachial artery [22, 34].

## Superficial brachial artery

Variations in the arterial pattern of the upper limb are quite common. Generally, they are encountered during routine dissections, and the superficial brachial artery (SBA) is one of the most popular variations. The frequency and branching pattern of the SBA vary among ethnic groups [35]. According to the literature, this variant can be seen in less than 1–25% of cases [7]. Usually at the proximal aspect of the BA, the median nerve (MN) lies lateral to the BA, then from the anterior but in described variation the BA courses superficially to the median nerve; this variant is called the SBA. However, SBA has many possible arterial variations within the upper limb and many terminologies. Another crucial impediment to classification is a separate analysis for the arm and forearm [7]. Rodríguez-Niedenführ et al. [29] indicate that the SBA represents a BA, which superficially descends in front of the MN instead of coursing deep to it. This variation at the level of the elbow joint branches into the RA and UA like normal BA [29]. When the SBA occurs, it may replace the main BA trunk completely and may be accompanied by an equally important, more or less important BA trunk running deep to the MN [25].

To understand the reason for the presence of SBA in adulthood as a morphological variability, it is necessary to refer to embryonic development. The SBA is a consistent embryonic vessel that plays a crucial role in the normal arterial morphogenesis of the upper limb [27]. Pursuant to the literature occurrence of SBA is a result of the persistence of more than one intersegmental cervical artery which does not degenerate but persists and can even enlarge its diameter [5, 18]. Rodríguez-Baeza et al. [27] mentioned that remain of SBA is determined by the hemodynamic predominance of some arterial segments during development [27, 35]. The frequency of occurrence of the SBA varies greatly. Referring to Rodríguez-Niedenführ et al. [28] frequency of occurrence of the SBA was 18 out of 192 cadavers (9.4%) with no statistically crucial differences between side or sex [7, 28]. In a Korean study, Yang et al. [35] observed the SBA artery in 37 extremities emerging from the axillary artery among 304 arms (12.2%). The incidence of SBA was not related to gender but was more frequent on the right, although insignificantly so [35]. McCormack et al. [21] found eight SBA during the dissection of 750 arms (1.07% of all examined limbs). In seven out of eight cases reported by McCormack et al. [21], the main trunk of the SBA arose from the BA. In the remaining case, the SBA originated from the AA [7, 21].

**Fig. 1:**
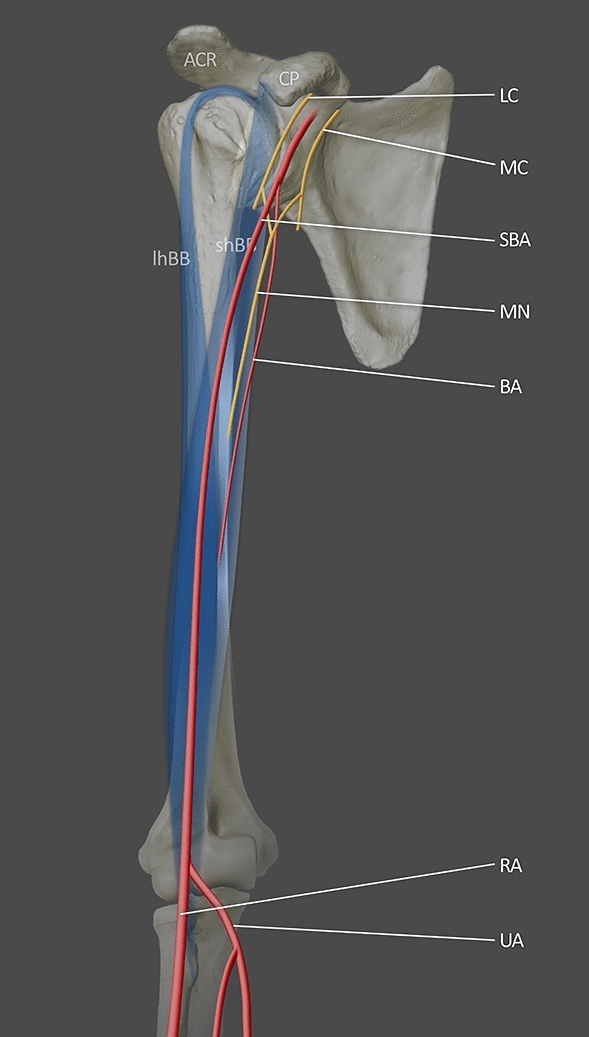
Illustration of type I SBA based on Yang et al. [35]. The SBA bifurcated into the RA and the UA. *LC* lateral cord; *MC* medial cord; *MN* median nerve; *BA* brachial artery; *SBA* superficial brachial artery; *RA* radial artery; *UA* ulnar artery

Yang et al. [35] distinguished three types of SBA in Korean cadavers. Type I gave off muscular branches to the biceps brachii and brachialis muscles then bifurcated into the RA and UA in the cubital fossa. The axillary artery became the (definitive) brachial artery to end as a collateral branch around the elbow joint. The frequency of Type I was 8.9%, i.e., 27 out of 37 all types of SBA. In this variant, the SBA may be more frequent than the definitive BA (Fig. 1).

**Fig. 2:**
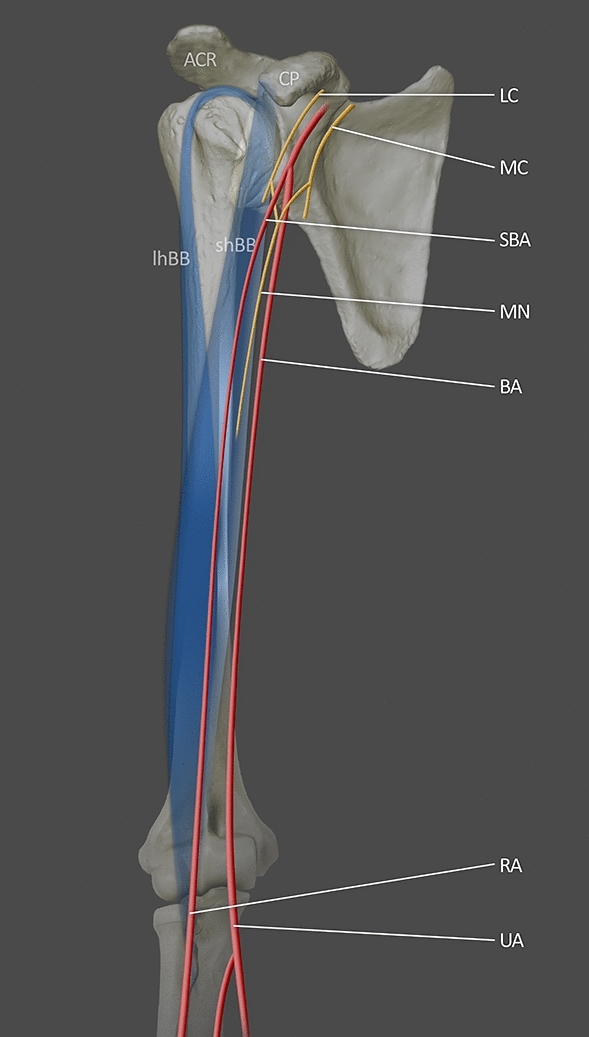
Illustration of type II SBA based on Yang et al. [35]. The SBA continued as the RA. *LC* lateral cord; *MC* medial cord; *MN* median nerve; *BA* brachial artery; *SBA* superficial brachial artery; *RA* radial artery; *UA* ulnar artery

The SBA may also form a parallel artery to the DBA. This Type II SBA is a variant that is continued as the radial artery in the forearm after branching off into muscular branches to the brachialis muscle and biceps brachii muscle. In the report, there were seven cases with a Type II SBA, which indicates an incidence of 2.3%. According to the classification proposed by Yang et al. [35] Type II SBA is a high origin of the radial artery, and it is the most frequent variation in Western populations. The frequency of Type II is 7% by Fuss et al. (“Type 5”) and 6% by Keen (“Type B”) [10, 17, 35] (Fig. 2).

**Fig. 3:**
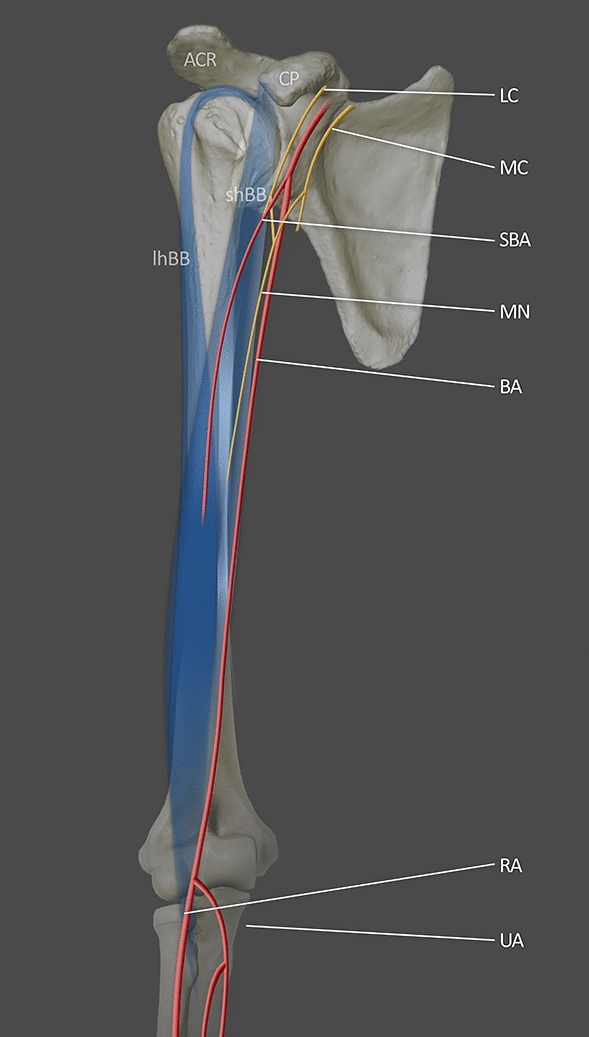
Illustration of type III SBA based on Yang et al. [35]. The slender SBA supplied the arm musculature and ended in the upper arm. *LC* lateral cord; *MC* medial cord; *MN* median nerve; *BA* brachial artery; *SBA* superficial brachial artery; *RA* radial artery; *UA* ulnar artery

Type III SBA was found in only three cases (1%). Its main role was to supply arm musculature. Type III is a small artery that ends in the arm; as such, this type does not participate in any arterial connection [35] (Fig. 3).

**Fig. 4:**
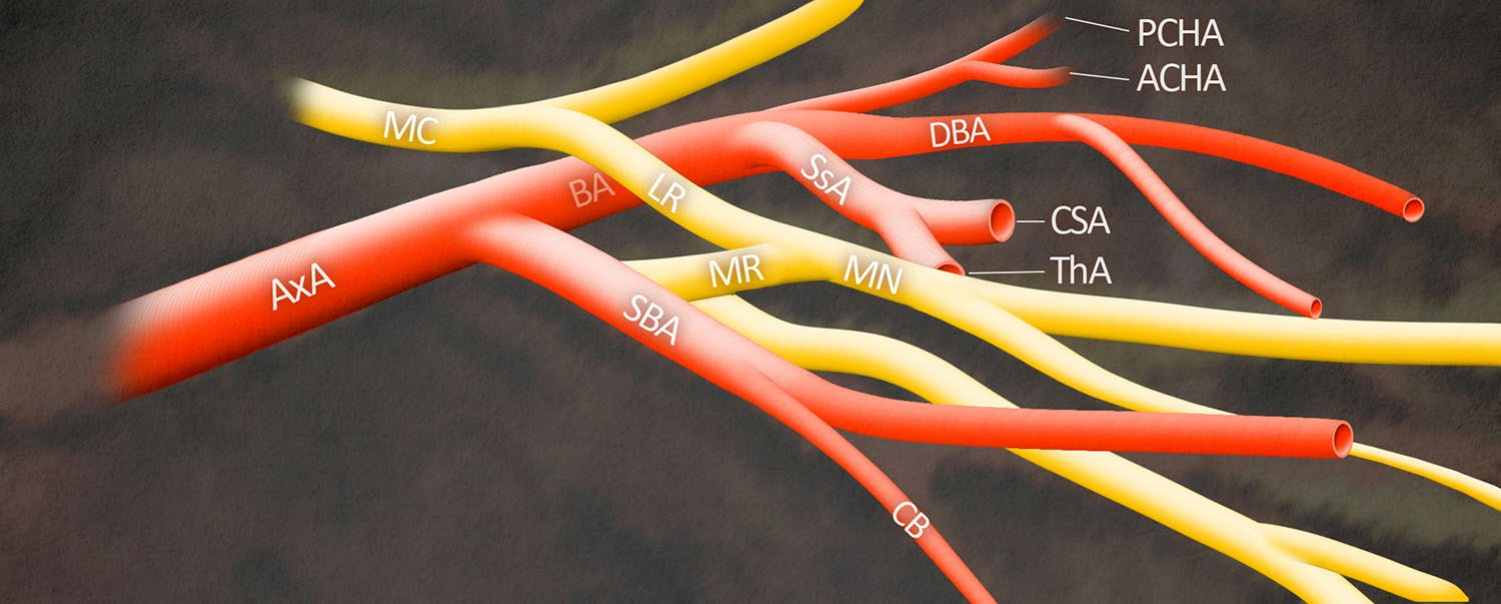
Anterior view of the left upper limb of a 75-year-old male Caucasian showing an SBA based on Nkomozepi et al. [25]. *MC* medial cord; *LR* lateral root; *MR* medial root; *MN* median nerve; *AxA* axillary artery; *BA* brachial artery; *SBA* superficial brachial artery; *CB* cutaneous branch; *SsA* subscapular artery; *CSA* circumflex scapular artery; *ThA* thoracodorsal artery; *DBA* deep brachial artery; *PCHA* posterior circumflex humeral artery; *ACHA* anterior circumflex humeral artery

One of the clinical implications resulting from the presence of the SBA is an entrapment neuropathy of the MN. It is directly related to the uncommon course of the BA [7]. MN entrapment syndromes have mostly been reported to occur at points between the wrist joints and the elbow joints, but when the MN or its roots entrap between the SBA and the AA idiopathic MN neuropathies may be triggered [19]. This ailment can be understood by analyzing the anatomical relationships between the nerves and arteries of the upper limb, as shown in Fig. 4. This figure shows the SBA originated from the AA which then descended superficial to the medial root of the MN, and this could represent a potential cause of neuropathy. Illustrated case of male Caucasian corresponds to type I SBA according to Yang et al. [35]. classification. However, in the study conducted by Nkomozepi et al. [25] the BA did not terminate as a collateral branch but rather ended as the DBA.

When the abnormal course of the SBA is a source of compression the MN pressure may cause ectopic stimulation of sensory fibers. This kind of situation could lead to motor disturbance, sensory neuropathy, and acute pain. Symptoms of MN compression arising from such variations are often confused with more common causes, such as carpal tunnel syndrome, pronator teres syndrome or even radiculopathy [5]. According to Liu et al. [19], one solution, excluding surgical intervention, is to reduce the compression of the SBA on the MN by conservative treatment based on muscle relaxation and acupuncture. The procedure appears to relieve the pressure of the surrounding soft tissue, which in turn helped to decrease the impingement of the SBA on the MN and return the neurological function to almost normal levels after two months of treatment [19].

## Brachioradial artery

**Fig. 5:**
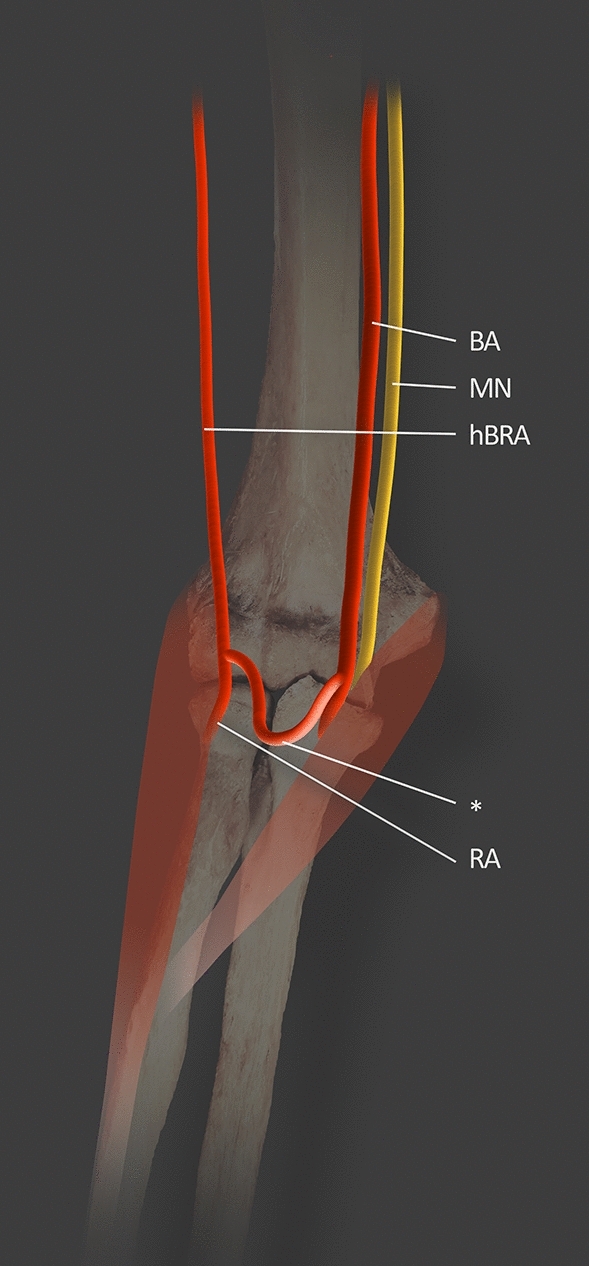
Dominant type of cubital crossover based on Haładaj et al. [12]. *BA* brachial artery; *hBRA* hypoplastic brachial segment of the brachioradial artery; *MN* median nerve; *RA* radial artery; * signify cubital connection

Mostly the BA bifurcates in the cubital fossa approximately 1.0 cm below the bend of the elbow, opposite the neck of the radius. This is the most common location through which the radial artery courses [12]. In some cases, the RA can display a high origin from the BA. There are also reported cases of emerging of the RA from the AA. Various authors have described and tried to classify this anatomical variation using terms like a high bifurcation of the BA or a double BA [12]. Yang et al. [35] describe the high origin of a radial artery as an SBA (type II SBA) which is continued as the radial artery in the forearm, as mentioned previously [35]. Rodríguez-Niedenführ et al. [28] propose the term “brachioradial artery” for the high origin RA. On its course, the brachioradial artery crosses the MN and adopts an anterior, superficial position to it along the arm. In the region of the antecubital fossa, the brachioradial artery passes posterior to the bicipital aponeurosis more often than anterior to it [28]. In studies conducted by Nasr [24], the brachioradial artery was observed in 8 out of 100 upper extremities (8% of all cases) from 30 adult male cadavers and 20 female cadavers. In seven cases, the source of RA was a BA; the RA took its course from the AA in only one case. The high origin of RA was observed in five right and three left upper extremities [24]. Haładaj et al. [12] also undertook brachioradial artery research. In the dissection of 120 upper extremities (65 male and 55 female limbs), the presence of a high origin of BA was found in 11 cases (9.2%) [12]. This variation was noticed in five female limbs and six male limbs, while the brachioradial artery arose from the BA in five female and four male upper limbs. In two remaining cases (both in male right limbs) the brachioradial artery branched off from the AA. Among 11 specimens, no significant difference was found with regard to the side: the BA occurred in six cases on the right side and in five cases on the left side. The abnormal high origin of RA was not predominant on the right or the left side [12]. Rodríguez-Niedenführ et al. [28] found 53 brachioradial arteries among 192 cadavers (53 out of 384 total upper limbs). This indicates an overall prevalence of 20.3%. In 25 cadavers, the brachioradial artery occurred unilaterally. In the case of the remaining 14 specimens, the brachioradial artery was located bilaterally. The brachioradial artery branched off from the BA in 40 cases and from the AA in 12 specimens. Unfortunately, in one case, the origin of the brachioradial artery could not be established [28]. The brachioradial artery has tendency to form a “cubital crossover” or “cubital connection” [12]: a structure formed in the cubital fossa by anastomosis between the normal trunk of the BA and the brachioradial artery [12]. Based on the diameter, certain types of cubital crossover can be distinguished: minimal, balanced, and dominant [12]. In a minimal cubital crossover, no differences can be seen between the diameter of the brachioradial artery in the region of the cubital connection and above it. The balanced type is characterized by a diameter slightly larger just below the anastomosis than in the brachial segment of the brachioradial artery. Haładaj et al. [12] report that in the balanced variant, the diameter of the brachioradial artery just below the anastomosis increased by 10.7% in a male left limb, 17.1% in a male right limb, and by 6% in a female limb. In turn, the dominant type is an anastomosis between the normal BA and brachioradial artery characterized by a greater diameter than the brachial segment of the brachioradial artery. Moreover, Rodríguez-Niedenführ et al. [28] noticed that when brachioradial artery is present the radial recurrent artery could start from it, from the DBA or from the cubital crossover; however, in most cases, the artery departed from the brachioradial artery [28] (Table 1, Fig. 5).

**Table 1:** Cases of brachioradial arteries according to Nasr [24], Haładaj et al. [12] and Rodríguez-Niedenführ et al. [28]

	Number of examined limbs	Number of brachoradial arteries	On the right	On the left	Origin from the BA	Origin from the AA
Nasr	100	8	5	3	7	1
Haładaj et al.	120	11	6	5	9	2
Rodríguez-Niedenführ et al.	384	53	30	23	40	12

**Fig. 6:**
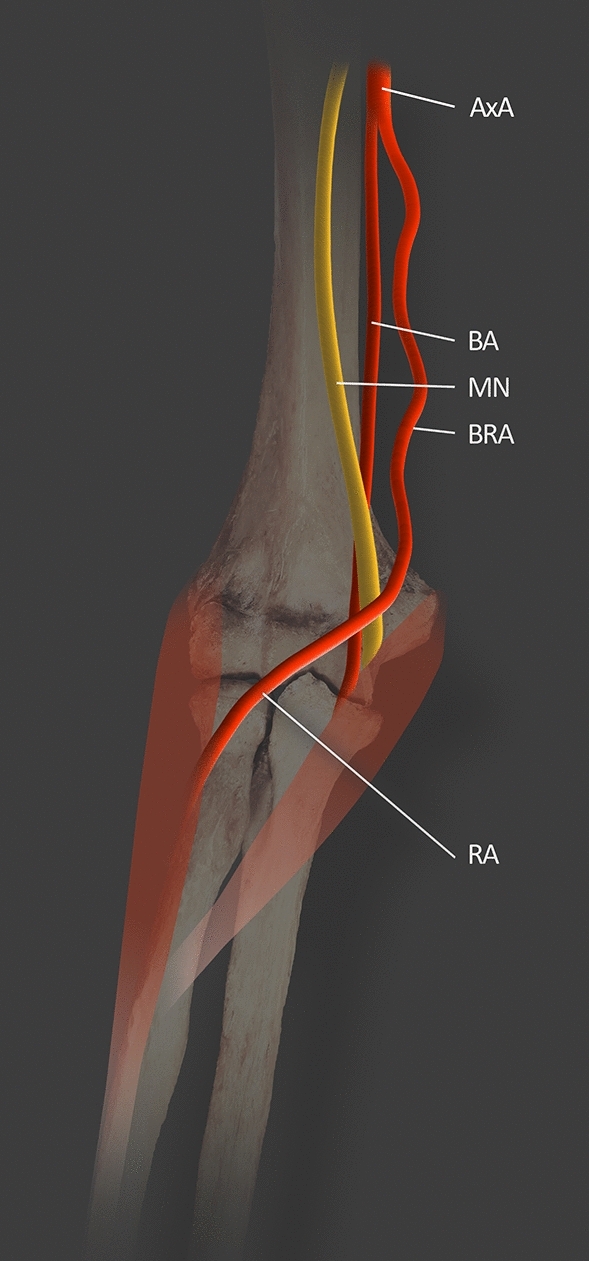
Left male upper limb with brachioradial artery based on Haładaj et al. [12]. The brachioradial artery descends superficially to MN. *MN* median nerve; *AxA* axillary artery; *BA* brachial artery; *BRA* brachioradial artery; *RA* radial artery

The presence of the brachioradial artery can cause similar effects to SBA due to entrapment neuropathy of the MN [24]. It is directly related to the very close proximity of the brachioradial artery and the MN (Fig. 6). The resulting neuropathies and treatments have been described above. Another clinical significance deriving from the atypical occurrence of the brachioradial artery is the possibility of confusing an artery with a vein when performing an intravenous injection. This may cause severe bleeding that poses a threat to the health and life of the patient [24]. Knowledge about this variation is necessary for proper angiography interpretation and performing surgical procedures on the upper limb [24]. The complicated arterial pattern created by the cubital crossover resulting from the occurrence of the brachioradial artery may increase the risk of iatrogenic injury during surgery in the region of the cubital fossa. A high origin of RA arising from the BA or AA may contribute to greater tortuosity of the vessel, which could increase the danger connected with the failure of transradial catheterization [12, 26]. The characteristic tortuous course of the brachioradial artery is given in Fig. 6; in such cases, catheterization is more complicated than usual. Variations of the RA are considered the second most frequent reason for failed transradial catheterization. The literature provides no unambiguous information as to their frequency; there are reports of frequencies from as low as 3% to as high as 20% [26].

## Accessory brachial artery

**Fig. 7:**
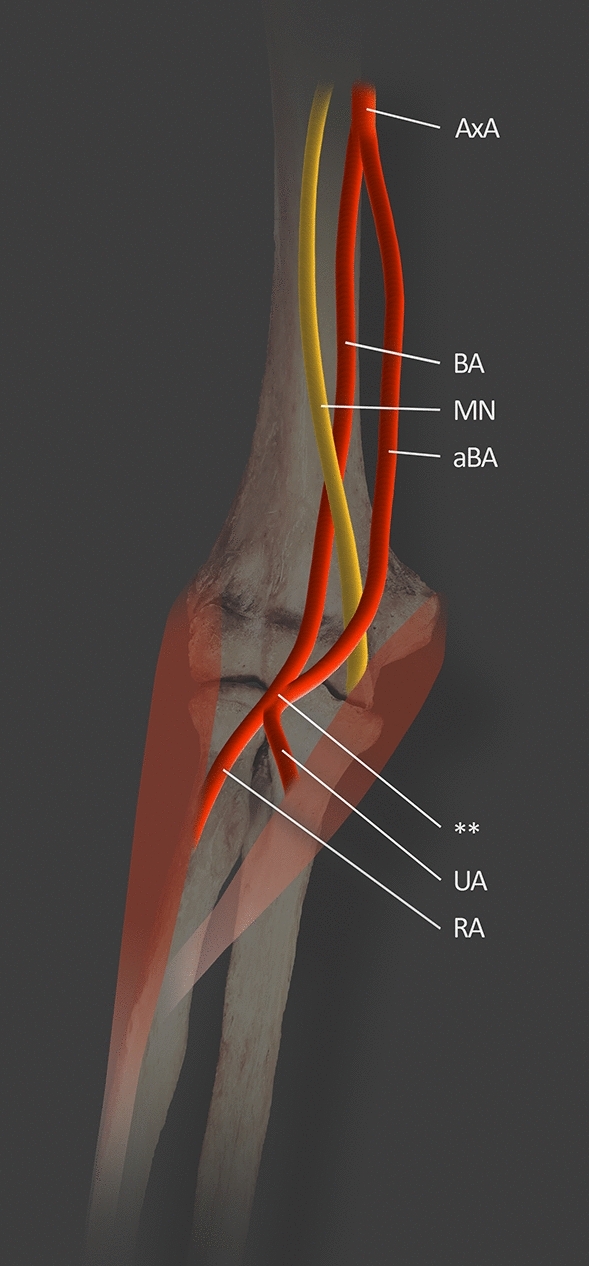
Right upper limb showing ABA and its reunion in the cubital fossa with the main BA based on Chakravarthi et al. [5]. *MN* median nerve; *AxA* axillary artery; *BA* brachial artery; *aBA* accessory brachial artery; *UA* ulnar artery; *RA* radial artery; ** accessory brachial artery reunion in the cubital fossa

Another morphological variation of the BA is the accessory brachial artery (ABA). This is a rare vascular abnormality located in the upper limb, with an incidence of <1% of cases [16]. The ABA is the artery originating from the BA or AA. The length of ABA ranges from 19 to 22 cm. After its course, the ABA rejoins the BA further along its distal course within the arm or in the region of the cubital fossa before giving off RA and UA [5, 16]. The ABA was first documented by Green [11] in the 1830s, in which the abnormal variant of ABA was described as a division of the AA into two vessels which unite at the fold of the arm. In addition, two BAs were identified, lying close to one another, and of equal size [11]. Rodríguez et al. [28] examined 192 cadavers (384 upper limbs) and described only one ABA (0.26% of all arms). This single variant protruded unilaterally on the right side in a male cadaver [28]. In studies conducted by Chakravarthi et al. [5] the ABA was noted in only eight female cadavers out of 70 cadavers (35 males and 35 females). Among these eight specimens, five demonstrated a unilateral ABA which merges with the main BA in the cubital fossa, while three demonstrated a bilateral ABA arising from the AA was found; in the latter three cases, the ABA did not rejoin with the main BA but continued as a superficial accessory ulnar artery. Kachlik et al. [16] identified a branch originating from the third part of the AA during dissection of 62 old women. This vessel began its course 2 cm distal to the lower margin of the pectoralis minor muscle. The ABA was located ventrally to the MN, then descended medially, accompanied by the ulnar nerve. During its course, the ABA gradually receded from the ulnar nerve towards the MN. In the distal part of the arm, the ABA rejoined the BA by a thin branch [16] (Table 2, Fig. 7).

**Table 2:** Cases of ABA reported by Rodríguez et al. [28] and Chakravarthi et al. [5]

Study conducted by	Number of examined upper limbs	Number of ABA	Male	Female
Rodríguez et al. [28]	384	1	1	0
Chakravarthi et al. [5]	140	11	0	8

The clinical relevance of the presence of an ABA concerns the danger during the catheterization through the radial or ulnar artery. When the long axis of the ABA meets the disto-proximal axis of the RA, the ABA might be misidentified, and the catheter could enter the ABA. As the ABA is two to three times narrower than the BA, the procedure will fail, also resulting in additional stress for the patient with an unusual arterial pattern [16]. Such proximity between the axis of the RA and the ABA may also contribute to iatrogenic damage.

## Absence of brachial artery

**Fig. 8:**
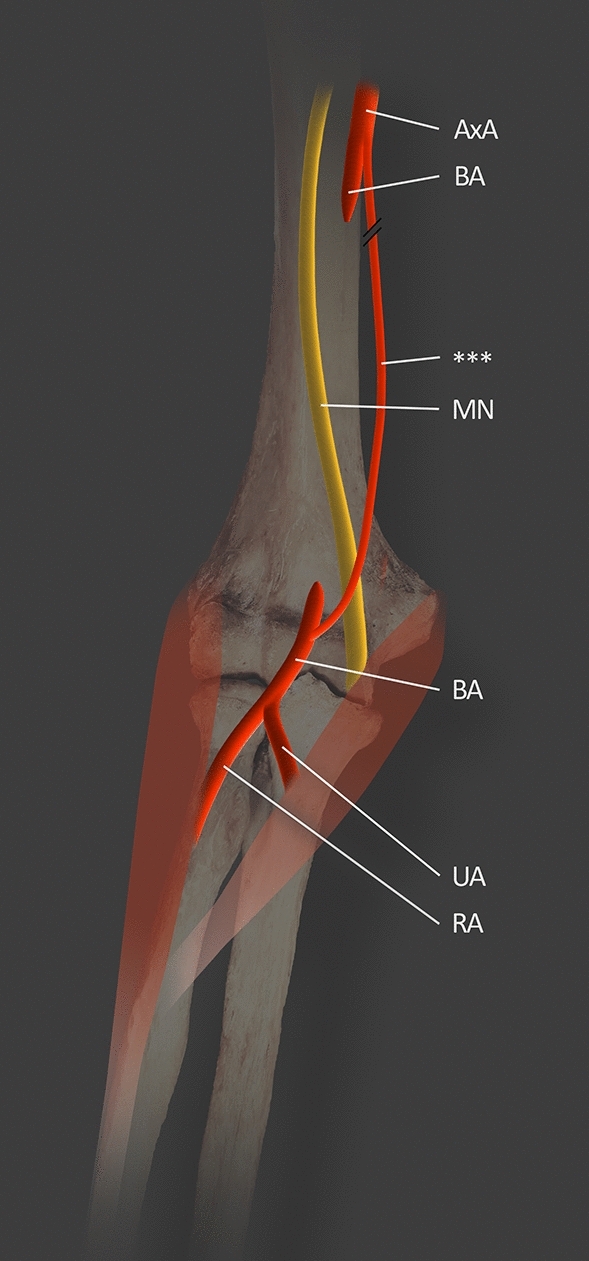
Absence of BA based on Ciervo et al. [6]. *MN* median nerve; *AxA* axillary artery; *BA* brachial artery; *UA* ulnar artery; *RA* radial artery; *** brachial artery reconstitution

A very interesting morphological variability of the BA is presented in a case report by Ciervo et al. [6] about a 63-year-old woman which demonstrated a total absence of the BA beyond its origin. The discovery was made during the surgical operation of the right upper limb. The patient needed a brachiocephalic arteriovenous fistula (AVF). No trauma or surgical intervention had occurred in the operated limb. An AVF is a direct communication between an artery and a vein (referring to described case report a connection between BA and cephalic vein) that results in blood being shunted between the two [2, 6]. When the surgeons exposed the cephalic vein with BA and created an anastomosis between these vessels, they noticed that the flow through the AVF was insufficient. The distal pulse was also not palpable. The BA field was then dissected, and surgeons observed the total absence of the BA beyond its origin. An unknown winding branch in the upper third of the arm, 3 mm in diameter, coursed between the triceps brachii and the coracobrachialis muscle. It is divided into an anterior and posterior branch on the level of the distal third of the arm. Due to the low flow, a brachio-brachial bypass graft was made with a reversed greater saphenous vein. This resulted in restoring the correct flow [6] (Fig. 8).

## The morphological variations of the brachial artery in relation to the external and internal diameter of the vessel

Other morphological variations of BA based on vessel diameter are indicated by Talalwah et al. [1]. In this study, 34 cadavers were dissected: 20 male and 14 female. The mean age of the specimens was 81 (men) and 75.9 years (women). All measurements were taken on the distal part of the artery before the bifurcation into its terminal branches, just below the origin of the DBA and its proximal part at the inferior border of the teres major muscle where the BA begins its course. The detailed results are presented in Table 3.

**Table 3:** External and internal diameter of BA based on Talalwah et al. [1]

Sample	Side	Mean External Diameter (mm)	Mean Internal Diameter (mm)
Male	*Right*		
	Proximal	7.55	7.12
	Middle	5.68	5.30
	Bifurcation	5.96	5.51
	*Left*		
	Proximal	7.24	6.82
	Middle	5.34	5.08
	Bifurcation	5.73	5.31
Female	*Right*		
	Proximal	5.89	5.48
	Middle	4.62	4.32
	Bifurcation	4.93	4.49
	*Left*		
	Proximal	5.77	5.17
	Middle	4.60	4.11
	Bifurcation	4.81	4.39
Combined	*Right*		
	Proximal	6.87	6.44
	Middle	5.24	4.90
	Bifurcation	5.54	5.09
	*Left*		
	Proximal	6.63	6.14
	Middle	5.04	4.68
	Bifurcation	5.35	4.93

Based on Al Talalwah et al. [1]

Based on the collected results, it can be concluded that on average the BA has a larger diameter in men than in women. In addition, the external and internal diameters of the examined vessel were not constant along its course: in both sexes, the BA had the largest external and internal caliber in its initial course [1].

**Fig. 9:**
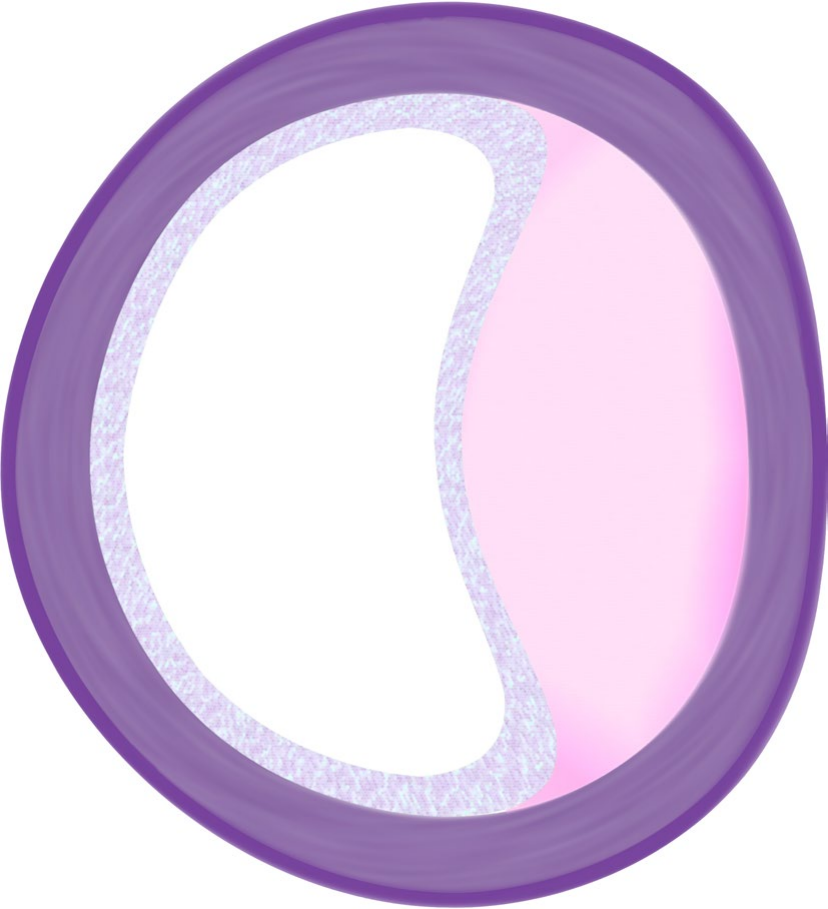
Advanced deep-seated lesion covered by fibrous cap within the BA based on Sorensen et al. [32]

The external and internal diameters of the BA are of great importance to vascular surgeons and clinicians. This knowledge can prove very useful when planning and creating arteriovenous fistula [1]. AVF is the preferred vascular access for hemodialysis, resulting in lower morbidity and mortality and lower costs than catheters or grafts [4, 15]. When analyzing the diameter of the BA, it is important to consider the effect of atherosclerotic disease. Atherosclerosis is a diffuse disease process characterized by the accumulation of lipids and fibrous elements in the large and medium-sized arteries such as the aorta or the coronary arteries [20]. Although the BA is similar in size to the major epicardial coronary artery, which supplies the heart muscle, it has rarely been studied for atherosclerosis. However, Sorensen et al. [32] suggest the possibility of atherosclerotic lesions within the BA. During autopsy in 52 examined subjects, atherosclerotic lesions of any grade were found within the BA in 39 cases. Additionally, a significant correlation was observed between the degree of atherosclerotic lesions in the brachial and carotid arteries. These findings indicate that atherosclerosis frequently occurs in the human BA [32] (Fig. 9).

Research conducted by Hamburg et al. [13] indicates the presence of maladaptive arterial remodeling in advanced obesity that was ameliorated by significant weight loss. An ultrasound study of 244 severely obese patients at baseline and 67 subjects who experienced weight loss over a year found higher BMI to be associated with larger BA diameter and lower shear stress [13].

## Conclusions

Knowledge about the morphological variations of the BA, its course, branching, and anastomoses is crucial while analyzing the angiograph, planning orthopedic, reconstructive and vascular procedures. Abnormal arterial patterns of the upper limb could disturb the evaluation of angiographic images during diagnosis and is more vulnerable to iatrogenic injury during different surgical procedures. A familiarity with the variation in the BA is also important both anatomically and clinically when understanding certain conditions, such as neuropathy.

## Data Availability

Please contact authors for data requests (Łukasz Olewnik, PhD—email address: lukasz.olewnik@umed.lodz.pl).

## References

[1] Al Talalwah W, Regassa D, Soames R (2015). Morphological feature of brachial artery and its clinical significance. J Morphol Sci.

[2] Beecher S, Alawy M, Elbakar A, Tubassam M (2014). Incidental discovery of a long standing arteriovenous fistula after thrombectomy for acute lower limb ischaemia. Int J Surg Case Rep.

[3] Breeland G, Alshuqayfi HA (2022) Anatomy, shoulder and upper limb, profunda brachii artery [updated 2021 Aug 25]. In: StatPearls [Internet]. StatPearls Publishing, Treasure Island, FL 31194382

[4] Bylsma LC, Gage SM, Reichert H, Dahl SLM, Lawson JH (2017). Arteriovenous fistulae for haemodialysis: a systematic review and meta-analysis of efficacy and safety outcomes. Eur J Vasc Endovasc Surg.

[5] Chakravarthi KK, Ks S, Venumadhav N, Sharma A, Kumar N (2014). Anatomical variations of brachial artery—its morphology, embryogenesis and clinical implications. J Clin Diagn Res.

[6] Ciervo A, Kahn M, Pangilinan AJ, Dardik H (2001). Absence of the brachial artery: report of a rare human variation and review of upper extremity arterial anomalies. J Vasc Surg.

[7] Clarke E, Mazurek A, Radek M, Żytkowski A, Twardokęs W, Polguj M, Wysiadecki G (2021). Superficial brachial artery—a case report with commentaries on the classification. Transl Res Anatomy.

[8] Duke J (2015). Anesthesia secrets.

[9] Epperson TN, Varacallo M (2021) Anatomy, shoulder and upper limb, brachial artery. In: StatPearls [Internet]. StatPearls Publishing, Treasure Island, FL. PMID: 30725830 30725830

[10] Fuss FK, Matula CW, Tschabitscher M (1985). Die arteria brachialis superficialis [The superficial brachial artery]. Anat Anz.

[11] Green PH (1830). An account of the varieties in the arterial system of the human body.

[12] Haładaj R, Wysiadecki G, Dudkiewicz Z, Polguj M, Topol M (2018). The high origin of the radial artery (brachioradial artery): its anatomical variations, clinical significance, and contribution to the blood supply of the hand. Biomed Res Int.

[13] Hamburg NM, Mott MM, Bigornia SJ, Duess MA, Kluge MA, Hess DT, Apovian CM, Vita JA, Gokce N (2010). Maladaptive enlargement of the brachial artery in severe obesity is reversed with weight loss. Vasc Med.

[14] Ichimura K, Kinose S, Kawasaki Y, Okamura T, Kato K, Sakai T (2017). Anatomic characterization of the humeral nutrient artery: application to fracture and surgery of the humerus. Clin Anat.

[15] Jennings WC, Sideman MJ, Taubman KE, Broughan TA (2009). Brachial vein transposition arteriovenous fistulas for hemodialysis access. J Vasc Surg.

[16] Kachlik D, Konarik M, Urban M, Baca V (2017). Accessory brachial artery: a case report, embryological background and clinical relevance. Asian Biomed.

[17] Keen JA (1961). A study of the arterial variations in the limbs, with special reference to symmetry of vascular patterns. Am J Anat.

[18] Konarik M, Knize J, Baca V, Kachlik D (2009). Superficial brachioradial artery (radial artery originating from the axillary artery): a case-report and its embryological background. Folia Morphol (Warsz).

[19] Liu J, Zhong K, Lin D (2020). Median nerve compression caused by superficial brachial artery: an unusual clinical case. J Int Med Res.

[20] Lusis AJ (2000). Atherosclerosis. Nature.

[21] McCormack LJ, Cauldwell EW, Anson BJ (1953). Brachial and antebrachial arterial patterns; a study of 750 extremities. Surg Gynecol Obstet.

[22] Mohammadzadeh MA, Mohammadzadeh M, Mohammadzadeh A, Herfatkar R, Mohammadzadeh V, Baghi I, Heydari H, Najafi S, Jalili M (2012). Arterial damage accompanying supracondylar fractures of the humerus. Trauma monthly.

[23] Moore KL, Dalley AF (1999). Clinically oriented anatomy.

[24] Nasr AY (2012). The radial artery and its variations: anatomical study and clinical implications. Folia Morphol (Warsz).

[25] Nkomozepi P, Xhakaza N, Swanepoel E (2017). Superficial brachial artery: a possible cause for idiopathic median nerve entrapment neuropathy. Folia Morphol (Warsz).

[26] Ostojić Z, Bulum J, Ernst A, Strozzi M, Marić-Bešić K (2015). Frequency of radial artery anatomic variations in patients undergoing transradial heart catheterization. Acta Clin Croat.

[27] Rodríguez-Baeza A, Nebot J, Ferreira B, Reina F, Pérez J, Sañudo JR, Roig M (1995). An anatomical study and ontogenetic explanation of 23 cases with variations in the main pattern of the human brachio-antebrachial arteries. J Anat.

[28] Rodríguez-Niedenführ M, Vázquez T, Nearn L, Ferreira B, Parkin I, Sañudo JR (2001). Variations of the arterial pattern in the upper limb revisited: a morphological and statistical study, with a review of the literature. J Anat.

[29] Rodríguez-Niedenführ M, Vázquez T, Parkin IG, Sañudo JR (2003). Arterial patterns of the human upper limb: update of anatomical variations and embryological development. Eur J Anat.

[30] Rosen RD, Bordoni B (2023) Embryology, aortic arch [updated 2023 Mar 6]. In: StatPearls [Internet]. StatPearls Publishing, Treasure Island, FL 31985966

[31] Shetty SD, Nayak BS, Madhav NV, Sirasanagandla SR (2012). The abnormal origin, course and the distribution of the arteries of the upper limb: a case report. J Clin Diagn Res.

[32] Sorensen KE, Kristensen IB, Celermajer DS (1997). Atherosclerosis in the human brachial artery. J Am Coll Cardiol.

[33] Tsoucalas G, Eleftheriou A, Panagouli E (2020). High bifurcation of the brachial artery: an embryological overview. Cureus.

[34] Usman R, Jamil M, Hashmi JS (2017). Management of arterial injury in children with supracondylar fracture of the humerus and a pulseless hand. Ann Vasc Dis.

[35] Yang HJ, Gil YC, Jung WS, Lee HY (2008). Variations of the superficial brachial artery in Korean cadavers. J Korean Med Sci.

[36] Zisquit J, Velasquez J, Nedeff N (2022) Allen test [updated 2021 Oct 2]. In: StatPearls [Internet]. StatPearls Publishing, Treasure Island, FL 29939593

